# Initiation of hepatic stellate cell activation extends into chronic liver disease

**DOI:** 10.1038/s41419-021-04377-1

**Published:** 2021-11-27

**Authors:** Vincent De Smet, Nathalie Eysackers, Vincent Merens, Mina Kazemzadeh Dastjerd, Georg Halder, Stefaan Verhulst, Inge Mannaerts, Leo A. van Grunsven

**Affiliations:** 1 Vrije Universiteit Brussel (VUB), Liver Cell Biology Research Group, Jette, Belgium; 2 KU Leuven, VIB Center for Cancer Biology, Leuven, Belgium

**Keywords:** Mechanisms of disease, Liver fibrosis

## Abstract

Activated hepatic stellate cells (aHSC) are the main source of extra cellular matrix in liver fibrosis. Activation is classically divided in two phases: initiation and perpetuation. Currently, HSC-based therapeutic candidates largely focus on targeting the aHSCs in the perpetuation phase. However, the importance of HSC initiation during chronic liver disease (CLD) remains unclear. Here, we identified transcriptional programs of initiating and activated HSCs by RNA sequencing, using in vitro and in vivo mouse models of fibrosis. Importantly, we show that both programs are active in HSCs during murine and human CLD. In human cirrhotic livers, scar associated mesenchymal cells employ both transcriptional programs at the single cell level. Our results indicate that the transcriptional programs that drive the initiation of HSCs are still active in humans suffering from CLD. We conclude that molecules involved in the initiation of HSC activation, or in the maintenance of aHSCs can be considered equally important in the search for druggable targets of chronic liver disease.

## Introduction

Chronic liver disease (CLD) relates to a spectrum of pathological states of the liver marked by inflammation, fibrosis and disturbed hepatocyte regeneration. When present over a prolonged period of time this can lead to the development of cirrhosis, liver failure and hepatocellular carcinoma (HCC). Causes of CLD include non-alcoholic fatty liver disease (NAFLD), viral hepatitis and alcohol abuse. To this day, CLD remains a global health issue accounting for an approximate 150.000 deaths each year in Europe associated with a significant economic burden [1–3]. Currently, targeting the underlying etiology is the only therapeutic option for CLD which is not feasible in many cases. Since the progression of fibrosis to cirrhosis in CLD is associated with an increased morbidity and mortality, developing antifibrotic therapies is an important yet unmet strategy for tackling CLD.

Liver fibrosis is marked by a pathological accumulation of extracellular matrix (ECM) proteins which are produced by hepatic stellate cells (HSCs). In a healthy liver, HSCs are quiescent and pericyte-like cells (qHSC), that reside in the space of Disse between parenchymal and endothelial cells. Here, their main function is to store retinyl esters and maintain a healthy ECM balance. During CLD, HSCs can differentiate into an activated, myofibroblast-like state (aHSC) and exert a proliferatory, contractile, fibrogenic, chemotactic and immunomodulatory phenotype [4–6]. Because of their pivotal role in liver fibrosis, removing or suppressing activated HSCs is an attractive strategy to treat CLD. However, no aHSC-targeting drugs that can efficaciously reduce fibrosis are available in the clinic. This is at least partially explained by our incomplete understanding of the HSC activation process and its maintenance.

The generally accepted model of how qHSCs differentiate into aHSCs proposes a two-step process: the first step is *initiation* which involves the priming of HSCs that sensitizes them to cytokines and other extracellular signals. The second step, *perpetuation*, involves the differentiation to their fully activated phenotype including the activation of ECM gene expression. This model implies that once liver fibrosis is established in patients with CLD, potential HSC-targeting therapies need to focus on the perpetuated phenotype since the initiation phase has long passed. Thus, current anti-aHSC therapeutic candidates mainly target characteristics of the aHSCs such as the activities of LOXL2 or TIMP1 [7, 8] which are required for scar tissue maturation. However, whether the initiation phase of HSC activation is still important after the onset of CLD is not known and has been neglected in the pursuit of new therapeutic candidates.

Motivated to identify new strategies to target HSCs, we analyzed the transcriptional programs present during the entire process of HSC activation, from initiation to perpetuation. We found that the transcriptional program in fully aHSC is already induced during HSC initiation and that the earliest transcriptional alterations during initiation extent into all stages of HSC differentiation, from the first liver injury into advanced liver disease in mice and humans. These results emphasize the importance of identifying gene regulator proteins that drive these transcriptional programs during the earliest stages of activation as a means for developing new HSC-targeting anti-fibrotic drugs.

## Materials and methods

### Ethical comments regarding animal usage

All animal experiments were approved by the Animal care and Use Committee of Vrije Universiteit Brussel (permits 14-212-4 and 18-212-1), the institution’s guidelines for the care and use of laboratory animals in research were strictly followed. Before and during experiments, all mice were housed in a controlled environment with free access to chow and water. For the duration of the experiment, animal welfare was followed-up daily and registered in a welfare diary. No randomization or blinding of mouse studies was performed.

### Experimental hepatotoxic liver injury

Male Balb/c mice were subjected to either acute or chronic hepatotoxic liver injury. For acute liver injury, mice aged 10–12 weeks were injected once with carbon tetrachloride (CCl_4_ - Sigma, Saint Louis, USA) and blood and livers collected for further analysis after 24 h (*n* = 5), 72 h (*n* = 4) and 7days (*n* = 5) (i.e., 1 day, 3 days and 7 days). For chronic liver injury, mice aged 8–10 weeks were injected with CCl_4_ on a semi-weekly basis for a total duration of 4 weeks. At three timepoints after this regimen (i.e. 1 day [*n* = 7], 3 days [*n* = 7] and 14 days [*n* = 6]), mice were sacrificed and blood and livers collected for further analysis. All mice were injected intraperitoneally with CCl_4_ diluted in mineral oil at a dosage of 0.5 µL per gram body weight. As control, non-treated (i.e. healthy) mice aging 10–12 weeks were used (*n* = 4). For HSC isolations, livers were perfused, processed and subjected to ultraviolet positivity-based sorting as previously published [9]. Isolated HSCs were immediately collected for RNA analysis without culturing. Liver injury was confirmed by alanine aminotransferase (ALT) measurements on plasma using a SPOTCHEM EZ SP-4430 (A.Menarini Diagnostics, Florence, Italy). Sample sizes were chosen based on RNA Sequencing requirements (2–4 biological repeats) [10]. More biological repeats (*n* = 5–7 total) were included for immunohistochemical analyses. Each repeat represents an individual experiment.

### Immunohistochemistry

Liver tissue was formalin fixed and embedded in paraffin. 5 µm sections were prepared on which either Picrosirius staining, or immunohistochemistry (IHC) was performed. For Picrosirius staining, sections were incubated for 60 min with Sirius Red solution containing Direct Red (Sigma) and Fast Green (Sigma) dissolved in saturated picric acid (Sigma). For IHC, antigen retrieval was performed by heating sections in citrate buffer (Dako, Santa Carla, USA) at 95° followed by peroxidase blocking (0.3% H_2_O_2_ in methanol) and permeabilization (0.05% Tween-20 [Sigma] in PBS). Next, sections were blocked with 2% BSA (Sigma) in PBS and incubated with primary antibody (α-SMA [ab32575] 1/600, Abcam, Cambridge, UK) overnight at 4°. After rinsing, sections were incubated with HRP-2^nd^ antibody conjugate (anti-rabbit, Dako). Peroxidase reactivity was visualized with 3,3’-diaminobenzidine (DAB, Sigma)/H2O2 and counterstained with hematoxylin (Sigma). Positively stained area was quantified using the machine-learning based Orbit Image Analysis software [11].

### Culture of mouse HSCs

Mouse HSCs from healthy male Balb/c mice aged 23–24 weeks were isolated by ultraviolet positivity-based sorting as previously published [12]. After isolation, a fraction of the cells was immediately collected for RNA analysis without culturing. The remainder was sorted directly into collagen coated (Corning, Bedford, USA) 24-well plates containing Dulbecco’s modified Eagle’s medium (DMEM - Lonza, Basel, Switzerland) supplemented with 10% fetal bovine serum (Lonza), 1% Penicillin-Streptomycine (Gibco, Carlsbad, USA) and 1% glutamine (Lonza) at 37 °C in a humidified atmosphere with 5% CO_2_. Culture medium was changed after 3 h and 24 h and cells were collected for RNA analysis after 3 h, 6 h, 12 h, 18 h, 24 h and 96 h. For every timepoint, 3 independent repeats were performed, and every independent repeat represents two technical repeats. During culture, bright-field images were taken with an Axiovert light microscope (Carl Zeiss, Oberkochen, Germany). Sample sizes were chosen based on RNA Sequencing requirements (2–4 biological repeats) [10].

### RNA extraction and sequencing and bioinformatic analysis

Total RNA was extracted from cells using the ReliaPrep RNA Cell Miniprep System (Promega, Madison, USA) according to supplier protocol and quality assessed using Bioanalyzer 6000. Only RNA samples with sufficient RNA quality were chosen. For in vivo samples, single-end sequencing was run on Illumina NextSeq 500 High with read length of 75 bp resulting in, on average, 29 M reads per sample. For in vitro samples, single-end sequencing was run on NovaSeq SP 100x6bp resulting in, on average, 7 M reads per sample. Quality control and trimming was performed using FastQC followed by mapping of the reads to the reference genome (Mus_musculus_GRCm38.p6) using STAR [13]. Assembly of genes and transcripts was performed using python package StringTie [14]. Once raw counts were generated, further analysis was performed in R using DESeq2 package [10].

### Downstream analyses of RNA sequencing

Once raw counts were generated, normalization of counts, determination of differentially expressed genes and principal component analysis (PCA) was performed using DESeq2 package in R environment [10]. Gene ontology (GO) analysis was performed using The Database for Annotation, Visualization and Integrated Discovery (DAVID) v6.8 [15]. Groups of differentially expressed genes with similar expression profiles were determined using DEGreport package [16]. Gene set enrichment analysis (GSEA) comparing two groups was performed using GSEA software v4.1.0 [17]. Multiple group GSEA was performed using Bubble GUM (GSEA Unlimited Map) [18]. For all GSEA analyses, phenotype permutation was chosen when at least seven samples were represented for each single phenotype, if not the case, gene set permutation type was chosen. Additionally, the false discovery rate (fdr) statistic with a threshold of 0.1 was used to determine whether a certain gene set was significantly enriched. Normalized enrichment score (NES) is shown as second statistic. To assess kinetics of transcriptional activity of gene sets across multiple conditions, Gene Set Variation Analysis (GSVA) was used. GSVA was performed in the R environment using “GSVA” package [19]. The default GSVA method with a Poisson distribution was run on integer values of RNASeq. To determine differential regulation of gene sets across conditions, a linear model was fit to the output GSVA enrichment scores and statistics were calculated by empirical Bayes moderation and false discovery rate adjustment using the limma package [20].

### Transcriptome datasets

Previously published transcriptome datasets were downloaded and imported into Rstudio (Supplementary Table 1). For microarray data, CEL files were read using either oligo [21] or affy [22] packages depending on array type and subjected to Robust Multichip Average (rma) algorithm for data normalization. For bulk RNA sequencing, data was analyzed as described above. For single cell RNA sequencing, all raw cell counts were normalized, scaled and clustered using principal component analysis using Seurat package [23]. Cell populations were identified using the same markers as in the original papers [24, 25]. Single cell signature scores were calculated using Single-Cell Signature Explorer [26].

### Statistical analysis

Where applicable, results are presented as either mean ± SEM or boxplots (median ± min to max values). All statistical analyses were performed in R environment. Normal distribution of datasets was assessed using Shapiro-Wilk normality test. No variance estimation was performed. Depending on data set distribution, following tests were performed: (i) One-way ANOVA with post-hoc Tukey HSD (more than two groups with normal distribution) or (ii) Kruskal–Wallis rank sum test with Dunn multiple comparisons (more than two groups with non-normal distribution). Results were considered significant when *p* < 0.05.

## Results

### Defining the transcriptional programs in HSCs during experimental liver fibrosis

**Fig. 1:**
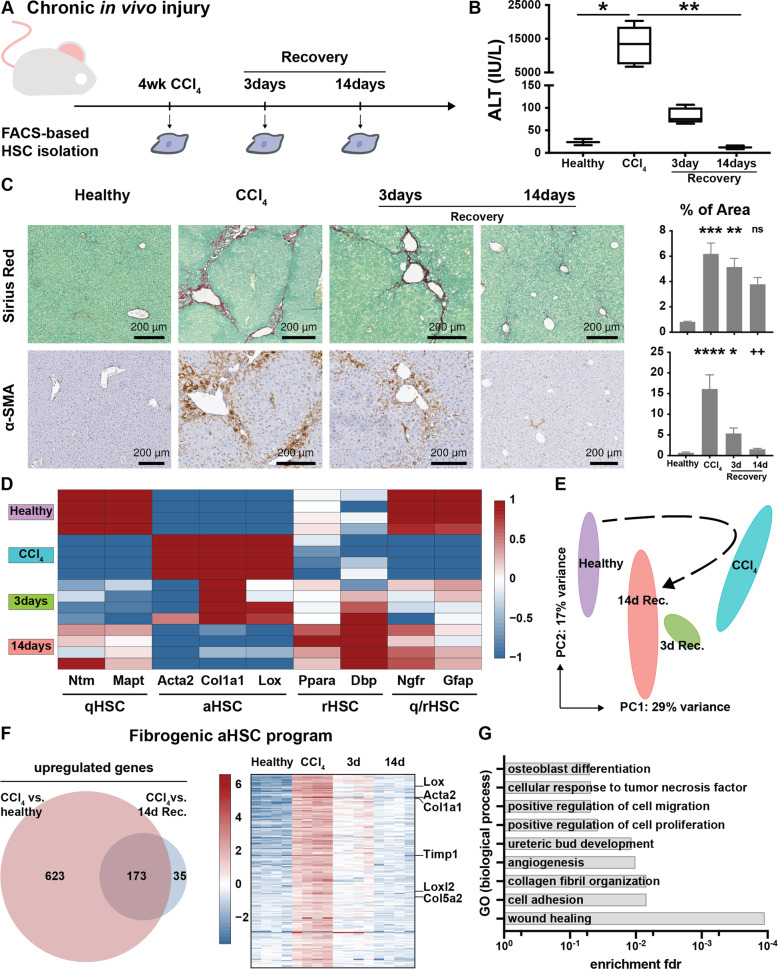
Generation of a fibrogenic aHSC program based on the CCl_4_ model of liver fibrosis and reversal. **A** Mice were injected with CCl_4_ for 4 weeks. HSCs were FACS-isolated at indicated timepoints and their RNA subjected to bulk sequencing. As a control, healthy (non-injected) mice were used. **B** ALT values indicate chronic liver injury and recovery. **C** Sirius red and α-SMA staining show fibrosis development and reversal. Barplots represent quantification of positive area. **D** Expression of phenotype specific genes during fibrosis induction and reversal. **E** Global transcriptional alterations of isolated HSCs by PC analysis. **F** Generation of the fibrogenic aHSC program. Differentially expressed genes in HSCs isolated from CCl_4_ treated mice compared to HSCs isolated from both healthy and recovered mice. Heatmap represents the defined 173 genes pinpointed as peak fibrosis genes. **G** GO analysis (biological process) of the fibrogenic aHSC program. ns *p* > 0.05, *p < 0.05, **p < 0.01, ***p < 0.001.

To achieve our goal of mapping transcriptional dynamics during different stages of HSC activation, we first set out to characterize the transcriptional program in fully activated (perpetuated) HSCs seen during peak fibrosis. As liver fibrosis in itself is a process of ECM remodeling, we aimed to define this program by selecting genes that are exclusively intertwined with ECM deposition. Hereto, we hypothesized that any transcriptional program active during fibrosis recovery cannot be directly responsible for ECM deposition and should thus be excluded when defining the perpetuated transcriptional program of HSCs. As such, we induced experimental liver fibrosis in Balb/c mice using CCl_4_ injections for 4 weeks and allowed additional groups of mice to recover for either 3 days or 2 weeks (Fig. 1A). As expected, CCl_4_ treatment resulted in increased ALT levels, collagen deposition and an increase in α-SMA positive cells. Recovery after termination of CCl_4_ was evidenced by reduced ALT levels, collagen deposition and amount of α-SMA positive cells (Fig. 1B, C).

Next, bulk RNASeq was performed on isolated HSCs. Principal component (PC) analysis showed a clear separation in transcription profiles of HSCs isolated from healthy livers from those isolated from fibrotic livers (healthy vs CCl_4_, Fig. 1E). Differential expression analysis revealed 796 upregulated genes in HSCs from fibrotic mice (4-weeks CCl_4_) compared to HSCs from healthy mice (Supplementary Table 2A). Genes included here constitute core matrisome genes such as collagens (e.g. *Col1a1*) and proteoglycans (e.g. *Vcan*) which are related to ECM deposition. We also noted upregulation of genes related to ECM digestion such as matrix metalloproteinases (e.g. *Mmp3*) and cathepsins (e.g. *Ctss*), indicative of the dynamic aspect of ECM turnover during fibrosis.

Upon recovery, HSC-transcriptional profiles shift back towards a more healthy-like profile as evidenced by PC analysis (3 day and 14 day recovery – Fig. 1E). We found 243 upregulated genes in HSCs from mice that had recovered for 2 weeks when compared to HSCs isolated from fibrotic mice (Supplementary Table 2B). Genes include known quiescent markers *Gfap*, *Ntm* and *Ngfr* as well as genes more specifically related to recovery such as *Ppara* and *Dbp*. Our findings support previous reports that HSCs have a similar, yet distinct phenotype in a recovering liver when compared to qHSCs (Fig. 1D, Supplementary Fig. 1A–D and Supplementary Table 2C, D).

Lastly, we noted that only 173 genes were significantly downregulated again after 2weeks of recovery while the remaining 623 genes maintained some level of upregulation during recovery. As such, these genes cannot be directly correlated to ECM deposition and were excluded from the transcriptional program of perpetuated HSCs (Fig. 1F). The remaining 173-gene set contained ECM genes such as collagens, *Loxl2* and HSC activation markers such as *Acta2*, but lacked many ECM digestion genes like cathepsins and metalloproteinases. Gene ontology (GO) analysis of these 173 peak fibrosis genes revealed enrichment of GO’s such as wound healing, collagen fibril organization, cell adhesion as well as angiogenesis (Fig. 1G). Here, we concluded that the 173 selected genes constitute a transcriptional program, hereafter referred to as fibrogenic aHSC program, compatible with the perpetuation phase of aHSCs found in experimental fibrotic livers (Supplementary Table 3).

### Early HSC activation is marked by fibrosis associated transcriptional dysregulation

**Fig. 2:**
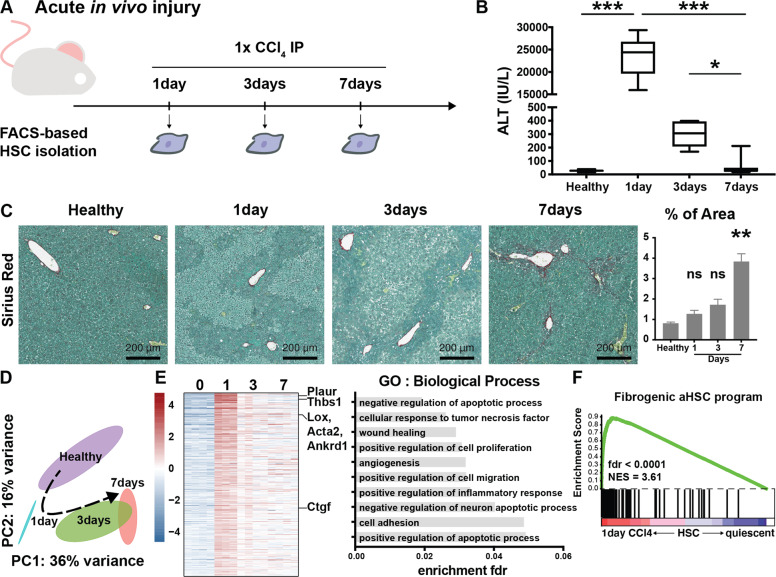
Fibrosis associated transcriptional alterations occur in HSCs during experimental acute liver injury. **A** Mice were injected once with CCl_4_ and HSCs were isolated at indicated timepoints and their RNA subjected to bulk sequencing. As a control, healthy (non-injected) mice were used. **B** ALT values indicate acute liver injury and recovery. **C** Sirius red staining shows necrotic areas after 1 and 3 days followed by collagen deposition after 7 days. Barplot represents quantification of positive area. **D** Global transcriptional alterations of isolated HSCs by PC analysis. **E** Heatmap of differentially upregulated genes 24 h after a single CCl_4_ injection compared to HSCs isolated from healthy mice with respective GO analysis. **F** GSEA of HSCs isolated 24 h after CCl_4_ compared to qHSC using the fibrogenic aHSC program defined in Fig.1. GSEA was performed with the “gene set” permutation type. ns *p* > 0.05, *p < 0.05, **p < 0.01, ***p < 0.001.

To investigate whether HSC initiation was marked by similar transcriptional changes, we induced acute liver injury in mice by means of a single injection of CCl_4_ and analyzed liver phenotypes after 1, 3 and 7 days (Fig. 2A). Increased ALT levels on day 1 were accompanied by necrotic areas throughout the liver parenchyma marking tissue injury. With increasing recovery time, ALT levels dropped and an increase in collagen deposition was seen after 7 days, indicative of liver regeneration (Fig. 2B, C).

Transcriptional profiles of isolated HSCs after acute injury show a division of profiles both over time (PC1) as well as over injury (PC2 – Fig. 2D). PC and differential expression analyses of the chronic and acute liver injury combined, suggests that HSCs do not seem to reach the same level of transcriptional dysregulation after 1 injection of CCl_4_, but HSCs recovered from a chronic CCl_4_ treatment for 3 days strongly resemble HSCs on day 7 after an acute CCl_4_ injury (Supplementary Fig. 2A–C, Supplementary Table 4). When comparing healthy HSCs with 24 h CCl_4_-HSCs, only few of the 424 upregulated genes were identified as fibrogenic genes in 4weeks CCl_4_-HSCs (e.g. Acta2 and Lox - Fig. 2E, Supplementary Table 5). GO analysis showed enrichment of many biological processes seen in HSCs isolated from fibrotic mice such as cell adhesion and wound healing (Fig. 1G and Fig. 2E). Gene set enrichment analysis (GSEA) on the acute liver injury set (24 h vs healthy) using the fibrogenic aHSC program defined above, showed a marked activation of this transcriptional program in 24 h CCl_4_-HSCs (Fig. 2F). These results indicate that, while at this stage a fibrosis phenotype is not evident, HSCs have already altered their transcriptional program towards a fibrogenic program in preparation for their future fibrogenic phenotype. These results are in line with the concept of HSC initiation.

### HSCs activate a fibrogenic transcriptional program 6 hours after in vitro culture

**Fig. 3:**
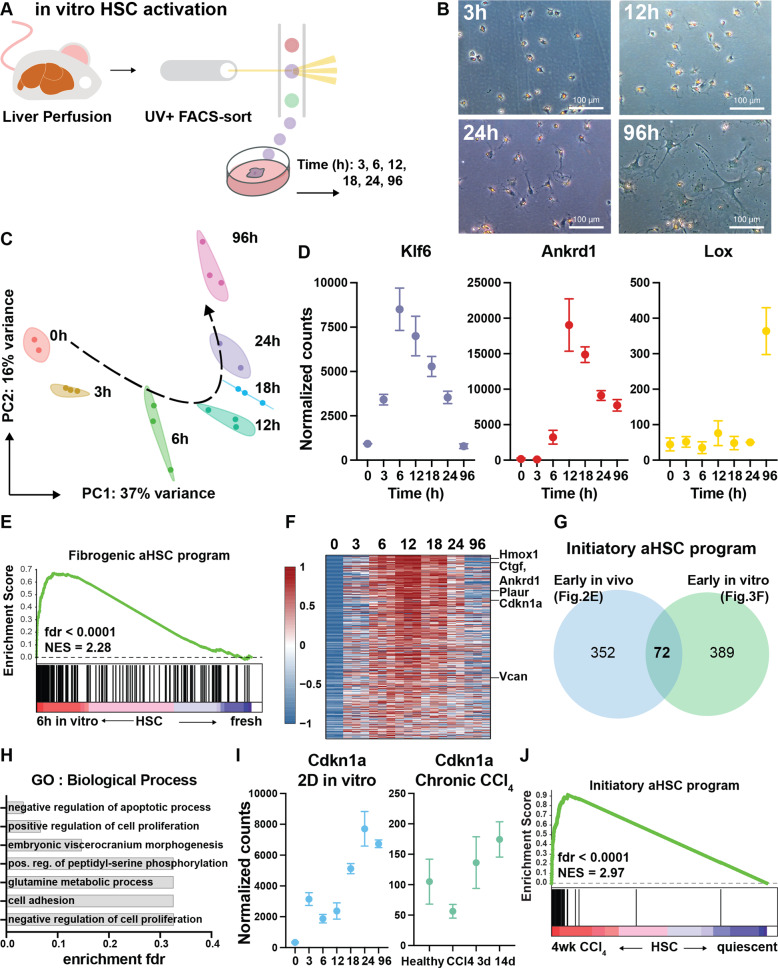
Defining an initiatory aHSC transcriptional program from in vitro and in vivo initiating HSCs. **A** After liver perfusion and NPF isolation, HSCs were sorted into culture dishes using FACS. At indicated timepoints, cells were collected for RNASeq. **B** Brightfield microscopic images of 2D cultured HSCs. **C** PC analysis of in vitro activated HSCs. **D** Gene expression of canonical initiation genes *Klf6* and *Ankrd1* and fibrogenic gene *Lox* during HSC activation. **E** GSEA of HSCs cultured for 6 h compared to freshly isolated HSCs using the fibrogenic aHSC program defined in Fig. 1. GSEA was performed with the “gene set” permutation type. **F** Heatmap of differentially upregulated genes in culture-activated mouse HSCs 6 h and 12 h after plating when compared to freshly isolated HSC. **G** To generate the initiatory aHSC program, the “early in vivo” (Fig. 2E) and “early in vitro” gene set were intersected (Fig. 4F). **H** GO analysis of the initiatory aHSC program. **I** Gene expression of *Cdkn1a* as example of dissimilarities between HSC initiation and perpetuation. **J** GSEA of HSCs isolated 24 h after 4week CCl_4_-induced liver fibrosis compared to qHSC using the initiatory aHSC program. GSEA was performed with the “gene set” permutation type.

Next, we wondered how quickly the fibrogenic aHSC program would be activated. To this end, we used a 2D in vitro model of HSC activation and analyzed the first 24 h of HSC activation with small intervals (Fig. 3A). Although in vitro HSC activation does not fully represent in vivo HSC activation, it allows for a better timed and controlled monitoring of the process [9]. HSCs from healthy mice were FACS-sorted directly into culture dishes and allowed to activate. While few phenotypical alterations were noted during the first 24 h, cells started to stretch and lose their lipid droplets by day 4, marking the start of myofibroblast differentiation (Fig. 3B).

In contrast to the lack of clear phenotypical alterations, PC analysis shows that major transcriptional changes take place during the first 24 h (Fig. 3C). Furthermore, differential gene expression analysis revealed that HSCs exert the biggest transcriptional alterations 12 and 18 h after start of the culture (Supplementary Fig. 3A). Next, we validated the time dependent induction of *Klf6*, *Ankrd1* and *Lox* (Fig. 3D). While the latter is a known marker for fully aHSCs, the first two genes are reported as HSC initiation markers [9, 27]. Interestingly, GSEA on the 6 h timepoint (vs freshly isolated HSCs) using the fibrogenic aHSC program showed significant enrichment even at this early stage (Fig. 3E). In conclusion, our results show that in their differentiation towards myofibroblasts, HSCs undergo major transcriptional alterations during the first hours of activation (i.e. initiation) to put a fibrogenic transcriptional machinery in motion.

### Perpetuated HSCs exert an initiating transcriptional program

During their initiation, HSCs upregulate distinct genes that do not per se maintain their elevated expression over time (e.g. *Klf6*). We thus hypothesized that a distinct transcriptional program is activated during initiation that is lost in the perpetuation phase. We defined this initiatory transcriptional program as genes conserved during both in vitro and in vivo initiation. Since the upregulation of canonical initiation genes *Ankrd1* and *Klf6* in vitro was most prominent at 6 h and 12 h in vitro, we defined consistently upregulated genes at these timepoints as in vitro initiation genes. (Fig. 3F). Next, we used the in vivo HSC initiation genes (upregulated in 24 h CCl_4_-HSCs—Fig. 2E) and intersected them with the in vitro genes. This led to a gene set of 72 genes, related to HSC initiation both in vivo and in vitro, that constitutes an initiatory aHSC transcriptional program (Fig. 3G—Supplementary Table 6). GO analysis of this program did not show any enrichment of perpetuation characteristics (Fig. 3H). In fact, only one GO (negative regulation of apoptotic process) reached the fdr significance level of 0.05. Of note, 39 genes (e.g. *Vcan*) of the initiatory aHSC program were also represented in the fibrogenic aHSC program (Supplementary Fig. 3B—Supplementary Fig. 3A, B). Other genes, like *Cdkn1a*, did not show upregulation during experimental CLD and thus are only related to HSC initiation (Fig. 3I). These results indicate that, next to a fibrogenic program, a distinct transcriptional program is activated during HSC initiation. Lastly, GSEA on perpetuated aHSC (4weeks of CCl_4_) showed a marked enrichment of the initiatory aHSC program even after 4weeks of CCl_4_, when fibrosis is evident (Fig. 3J). In conclusion, the HSC initiation and fibrogenic transcriptional programs presented here show distinct features and are both active as early as 6 h in vitro and after 4 weeks of CCl_4_ in vivo.

### Transcriptional dysregulation during stages of HSC activation

Since we had shown that the transcriptional programs induced at HSC initiation extend into CLD, we wanted to get an overview of the transcriptional programs driving HSCs during different stages of experimental liver disease. To this end, we first performed GSVA on the HSC RNASeq data presented in this manuscript, representing initiation (acute liver injury and in vitro—Fig. 2 and Fig. 3) and fibrosis and recovery (chronic liver disease—Fig. 1). For comparison, we selected gene sets corresponding to quiescent-, activated- and reverted HSCs (Supplementary Fig. 4A) and their functional characteristics (UPR, YAP, collagen metabolism and apoptosis—Table 1).

**Table 1:** Selected gene sets correlating with HSC phenotypes and their functional status.

HSC phenotype	Gene Set	Study	Synopsis
Quiescent HSC	**qHSC Zhang**	Zhang et al. (2016)^36^	Genes specifically expressed by HSC, higher in quiescent HSC vs. activated
Initiation associated HSC	**YT TEAD**	Zanconato et al. (2015)^37^	YAP/TAZ/TEAD direct target genes
	**YT ECM**	Meng et al. (2018)^38^	ECM-responsive genes regulated by ﻿the Hippo pathway
	**UPR**	Hallmark_Unfolded_Protein_Response	Genes up regulated during unfolded protein response; a cellular stress response related to the endoplasmic reticulum.
	**Initiatory aHSC**	This manuscript	Overlap in vivo activated (24 h) and in vitro activated (6 h and 12 h) genes
Fibrosis associated HSC	**aHSC Zhang**	Zhang et al. (2016)^36^	Genes specifically expressed by HSC, higher in activated HSC vs. quiescent
	**Fibr. aHSC**	This manuscript	Genes upregulated in activated HSC after 4week CCl_4_ treatment compared to healthy and recovered HSC
	**Coll. organiz**.	Go_Collagen_ Fibril_Organization	Any process that determines the size and arrangement of collagen fibrils within an extracellular matrix
Reversal associated HSC	**Coll. Catabolism**	Go_Collagen_ Catabolic_Process	The proteolytic chemical reactions and pathways resulting in the breakdown of collagen in the extracellular matrix, usually carried out by proteases secreted by nearby cells
	**Apoptosis**	Hallmark_ Apoptosis	Genes mediating programmed cell death (apoptosis) by activation of caspases
	**rHSC Troeger**	Troeger et al. (2012)^29^	Genes upregulated in reverted HSC 45days after last CCl_4_ injection when compared to quiescent HSC

**Fig. 4:**
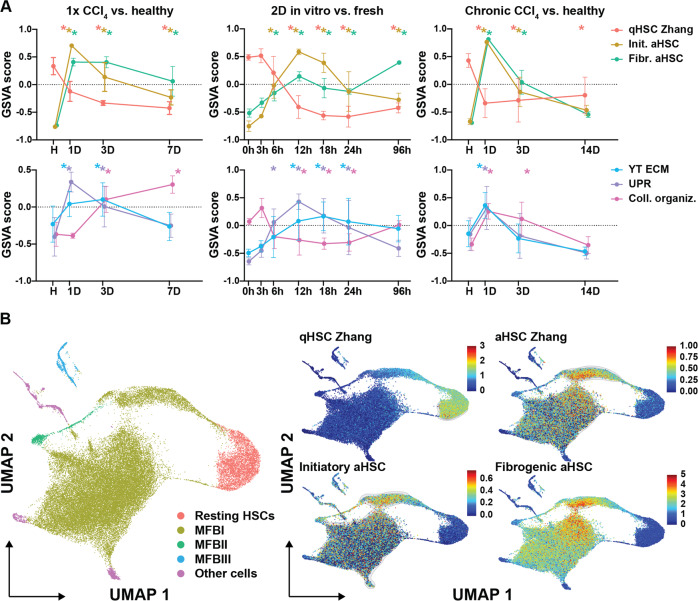
Transcriptional landscape during HSC initiation, perpetuation and recovery in experimental models of fibrosis. **A** GSVA on different models of HSC activation using the gene sets defined in Table 1 and bulk RNAseq data from this study. Left panels: 1x CCl_4_. Middle panels: in vitro HSC activation. Right panels: 4 weeks CCl_4_ and recovery. For GSVA, the gsva method with a Poisson distribution was run on integer values of RNASeq. Differential activity of gene sets was determined by fitting a linear model to the output GSVA enrichment scores. Statistics were calculated by empirical Bayes moderation and fdr adjustment. * fdr < 0.05 compared to control condition (healthy, 0 h and healthy respectively). **B** scRNASeq analysis of mouse HSCs in experimental model of chronic liver disease (3 weeks CCl_4_) [24]. The identified MFB clusters are: (i) MFBI: ECM production, (ii) MFBII: inflammatory mediator production and (iii) MFBIII: cell division. Left panel shows UMAP dimension reduction and determination of cell clusters based on published criteria. Smaller panels indicate Single-Cell Signature Score for indicated HSC gene sets (Table 1). Blue color represents low expression and increasing expression levels are green to red as shown by the legend.

During initiation, HSCs quickly lose their quiescent transcriptional program (qHSC Zhang) and activate others (aHSC Zhang, initiatory- and fibrogenic aHSC programs) that extend into CLD (CCl_4_) (Fig. 4A, Supplementary Fig. 5A). Early response events such as YAP downstream signaling (YT TEAD and YT ECM) and UPR, known to be activated during HSC initiation [9, 28], also extend into CLD (Fig. 4A, Supplementary Fig. 5A). However, these events seem to have a more transient aspect as they lose their enrichment both 1week after acute liver injury and 3days after chronic liver injury. In contrast to these early alterations, collagen turnover (collagen fibril organization and collagen catabolic process) is not induced during initiation (1day) and is only activated 3days after an acute liver injury and during CLD (Fig. 4A, Supplementary Fig. 5A). Recovery from CLD is marked by a loss of aHSC programs and a shift from collagen turnover towards collagen catabolism after 2 weeks of recovery. Furthermore, we noted that the reverted HSC transcriptional program (rHSC Troeger [29]) is the only HSC program still active after two weeks of recovery but is also activated during HSC initiation and CLD (Fig. 4A, Supplementary Fig. 5A). Additionally, our 2D timepoints showed that the initiatory and fibrogenic aHSC programs are both induced prior to the loss of the quiescent transcriptional program (Fig. 4A). Of note, collagen organization showed reduced transcriptional activity and the reverted HSC transcriptional program was not activated in in vitro cultures (Fig. 4A, Supplementary Fig. 5A).

Next, we verified our results in two independent transcriptome data sets of HSCs isolated from experimental models of liver disease (CCl_4_ and BDL, CCl_4_ and NASH) by performing GSEA [30, 31]. Similar to our chronic CCl_4_ injury data, we noted enrichment of the YAP/TAZ, initiatory, fibrogenic, aHSC Zhang, collagen turnover and rHSC sets. In all conditions, HSCs had lost the quiescent HSC program (Supplementary Fig. 5B). Here, we can conclude that fibrogenic events at the transcriptional level happen at very early stages during HSC activation and that transcriptional events during HSC initiation extend into chronic disease.

### Activated HSCs exert both initiatory and fibrogenic programs at the single cell level

Next, we considered the possibilities of both a “sequential” model, where HSCs with an active initiatory program transition into those with an active fibrogenic program, or a “simultaneous” model where both programs are active in the same cells. To distinguish between these possibilities, we analyzed single cell (sc) RNASeq data from HSCs isolated from mice treated with CCl_4_ for 3 weeks [24]. Our analysis shows that the fibrogenic aHSC program was widely enriched in the myofibroblast (MFB) type I cluster cells when compared to both the qHSCs (resting) and the MFBII and MFBIII populations (Fig. 4B). Furthermore, the initiatory aHSC program was only clearly enriched in a subcluster of MFBI and to a lesser extend in the other MFB populations (Fig. 4B). This scRNAseq analysis suggests that initiatory and fibrogenic programs are not necessarily sequential as both programs can be active in the same HSC during experimental CLD.

### Scar associated mesenchymal cells in human chronic liver disease exert both initiatory and fibrogenic transcriptional programs

**Fig. 5:**
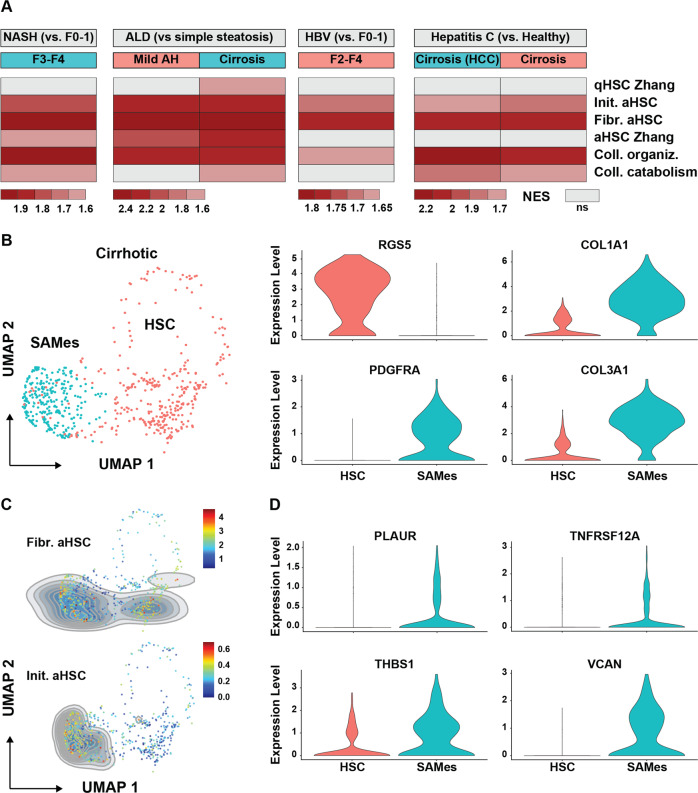
Scar associated mesenchymal cells in human CLD maintain characteristics of HSC initiation. **A** GSEA of human livers using gene sets defined in Table 1. Color legend indicates normalized expression score while a gray color indicates no enrichment in any direction for the indicated gene set. Analysis was performed by using Bubble GUM (GSEA Unlimited Map). When at least seven samples were represented for each single phenotype, “phenotype” permutation was chosen. Otherwise “gene set” permutation type was chosen. **B** scRNAseq analysis of HSC and SAMes in cirrhotic patients [25]. Left panel shows UMAP dimension reduction and determination of cell clusters based on published criteria. Smaller panels indicate individual gene expression of *RGS5*, *PDGFRA*, *COL1A1* and *COL3A1*. **C** Single-Cell Signature Score on HSCs and SAMes for indicated HSC gene sets (Table 1). Blue color represents low expression and increasing expression levels are green to red as shown by the legend. **D** Individual gene expression of genes represented in the initiatory aHSC program that are differentially upregulated in SAMes when compared to HSCs.

Globally, NASH, alcoholic liver disease (ALD) and hepatis B (HBV) and C (HCV) viral infections are the major causes of CLD and cirrhosis [32]. GSEA on transcriptome data of total liver samples collected from CLD patients showed enrichment of both initiatory and fibrogenic aHSC programs (Fig. 5A). To determine whether the initiatory and fibrogenic programs are also active in the same (activated) HSC during human CLD, we analyzed scRNASeq data generated from cirrhotic patients as reported by Ramachandran et al. [25]. In their manuscript, several mesenchymal cell types such as HSCs (RGS5 + ) and scar associated mesenchymal cells (SAMes – PDGFRα + ) are distinguished [25]. We confirmed the clustering of HSCs and SAMes based on *RGS5* and *PDGFRA* expression and show that SAMes have enriched expression of collagens (Fig. 5B). While the fibrogenic aHSC program is active both in HSCs and SAMes, we found that the initiatory program was exclusively represented in SAMes (Fig. 5C) and that genes such as *VCAN*, *THBS1* and *TNFRSF12A* are partly responsible for this enrichment as they are significantly upregulated in SAMes when compared to HSCs (Fig. 5D). Combined, these results show that during chronic- and end-stage human liver disease, highly fibrogenic mesenchymal cells maintain characteristics of HSCs that are initiating activation.

## Discussion

The model of initiation and perpetuation has provided us with a framework for mechanistic events occurring in sequence during HSC activation. Initiation was defined as rapid changes in gene expression and phenotype that render the cells responsive to cytokines and other local stimuli in the subsequent perpetuation phase [27]. We used a transcriptional approach to investigate the transcriptional alterations during all stages of HSC activation and provide evidence that there is no clear distinction between the initiation and perpetuation phases at the transcriptional level. We show that a fibrogenic transcriptional program seen during peak fibrosis occurs in initiatory HSCs (6 h in culture) despite a lack of phenotypical alterations or clear changes in commonly used fibrosis markers. Conversely, we found that during chronic and end-stage human liver disease, highly fibrogenic mesenchymal cells maintain a transcriptional program of initiating HSCs.

Based on transcriptional dynamics during fibrosis and recovery, we pinpointed a transcriptional program consistent with peak fibrosis in mouse HSCs that can identify highly active, ECM-producing HSCs. Nonetheless, this program was highly enriched in HSCs 24 h after acute liver injury and 6 h after in vitro culture. These results suggest that qHSCs already contain gene regulatory proteins required for a profibrogenic response at the transcriptional level. How qHSCs are able to swiftly respond to acute injury and induce a fibrogenic program in a matter of hours remains to be elucidated. Further epigenetic and gene regulatory analyses are required to map chromatin accessibility and putative regulators in initiatory and fibrogenic phases.

In humans, reports on the role of HSCs during acute liver failure (ALF) are very scarce and very few mechanistic studies have been performed. Currently, it is assumed that HSCs play a protective role during ALF, where increased TIMP-1 serum levels and increased liver stiffness were observed [33]. In the light of ALF, where liver function can be lost in a matter of hours, a rapid fibrogenic response to safeguard liver integrity seems more plausible than requiring the HSC to be primed before any fibrogenic response can take place.

Despite the similarities in fibrogenic response between HSC initiation and HSCs during CLD, we did note some distinct features during the earliest phases of HSC activation. Surprisingly, this *initiatory aHSC* transcriptional profile remains active 7days after an acute liver injury, and even in CLD in mice and humans. Analysis of scRNASeq revealed that in the ECM producing mouse aHSCs with the highest enrichment in fibrogenic genes, the *initiatory aHSC* program was enriched as well. More importantly, scRNASeq analysis of human cirrhotic livers showed that SAMes had higher enrichment of the initiatory program than HSCs that are not present in scar tissue. These findings suggest that initiatory events take place in CLD further building on a nuanced view of transcriptional dynamics in HSC activation. Whether the simultaneous activity of these programs in highly activated HSCs could indicate the dependency of the fibrogenic program on the initiatory program remains to be elucidated.

In HSCs isolated from livers recovering from injury, we noted a swift and sustained enrichment of the reversal set defined by Troeger et al. [29] in both acute and chronic liver injury. This gene set was based on a comparison of qHSC to HSCs isolated from mice that had recovered from chronic injury for 45 days. These results could indicate that activation of this gene set represents a transcriptional signature of current or previous activation, rather than a gene set consistent with recovery alone. Our results on recovery from chronic liver injury are in line with scRNASeq data from Rosenthal et al. that showed that HSCs fail to upregulate certain quiescence-related gene regulatory proteins and thus are not able to regain a fully quiescent phenotype during recovery [34] and thereby might show an enhanced response to recurrent injury [29]. Furthermore, our results support a transcriptional framework for inactivated HSCs, where, once fibrogenic transcriptional machinery is put in place, HSCs are permanently hinged towards a profibrogenic state even in the absence of injurious stimuli.

From our point of view, our novel conceptual insights also have therapeutic implications. In ALD, NASH, HBV and HCV, the extent of liver fibrosis has major prognostic implications which makes anti-fibrotic therapies one of the main goals to tackle CLD [7, 8, 35]. Current therapeutic targets mainly pertain to inhibiting or reverting the perpetuated state of HSCs at the transcriptional level (e.g. PPARγ agonists) or reducing the inflammatory (e.g. CCR2/CCR5 antagonists) and fibrogenic (e.g. LOXL2 or TIMP1 antagonists) phenotype of HSCs [7, 8]. Since we show here that transcriptional programs linked to HSC initiation still occur during CLD, we conclude that initiatory events are not exclusive to the onset of liver disease and merit further investigation in the pursuit of novel therapeutic targets.

In conclusion, initiation and perpetuation of HSC activation at the transcriptional level are not sequential processes and occur during acute liver failure, throughout CLD and even into cirrhosis. To enlarge the pool of druggable targets of chronic liver disease, one can consider molecules involved in the initiation of HSC activation or the maintenance of activated HSCs as equally important.

## Supplementary information

Supplemental documents

Supplementary Table 2

Supplementary Table 3

Supplementary Table 4

Supplementary Table 5

Supplementary Table 6

## Data Availability

Raw and processed sequencing data files have been uploaded to the Gene Expression Omnibus (accession no. GSE173920 and GSE176042).

## Code availability

All analyses requiring coding were performed in R environment. Any code can be made available upon author request.
