# Supplementary material for: Initiation of hepatic stellate cell activation extends into chronic liver disease
Source: Cell Death Dis. 2021 Nov 27;12(12):1110. doi: 10.1038/s41419-021-04377-1 (PMC8627507; doi:10.1038/s41419-021-04377-1)
Supplement: Supplementary file 1 — Supplemental documents [file 41419_2021_4377_MOESM1_ESM.docx]

#### Supplementary data to

**Initiation of hepatic stellate cell activation extends into chronic liver disease**

#### Authors

Vincent De Smet, Nathalie Eysacker, Vincent Merens, Mina Kazemzadeh Dastjerd, George Halder, Stefaan Verhulst, Inge Mannaerts, Leo A. van Grunsven

#### Table of Contents

Supplementary Figures…………………………………………………………........……..2

Supplementary Tables….……………………………………………………….................8

Supplementary References……………………………………………………........…….12

**Supplementary Figures**

Supplementary Figure 1


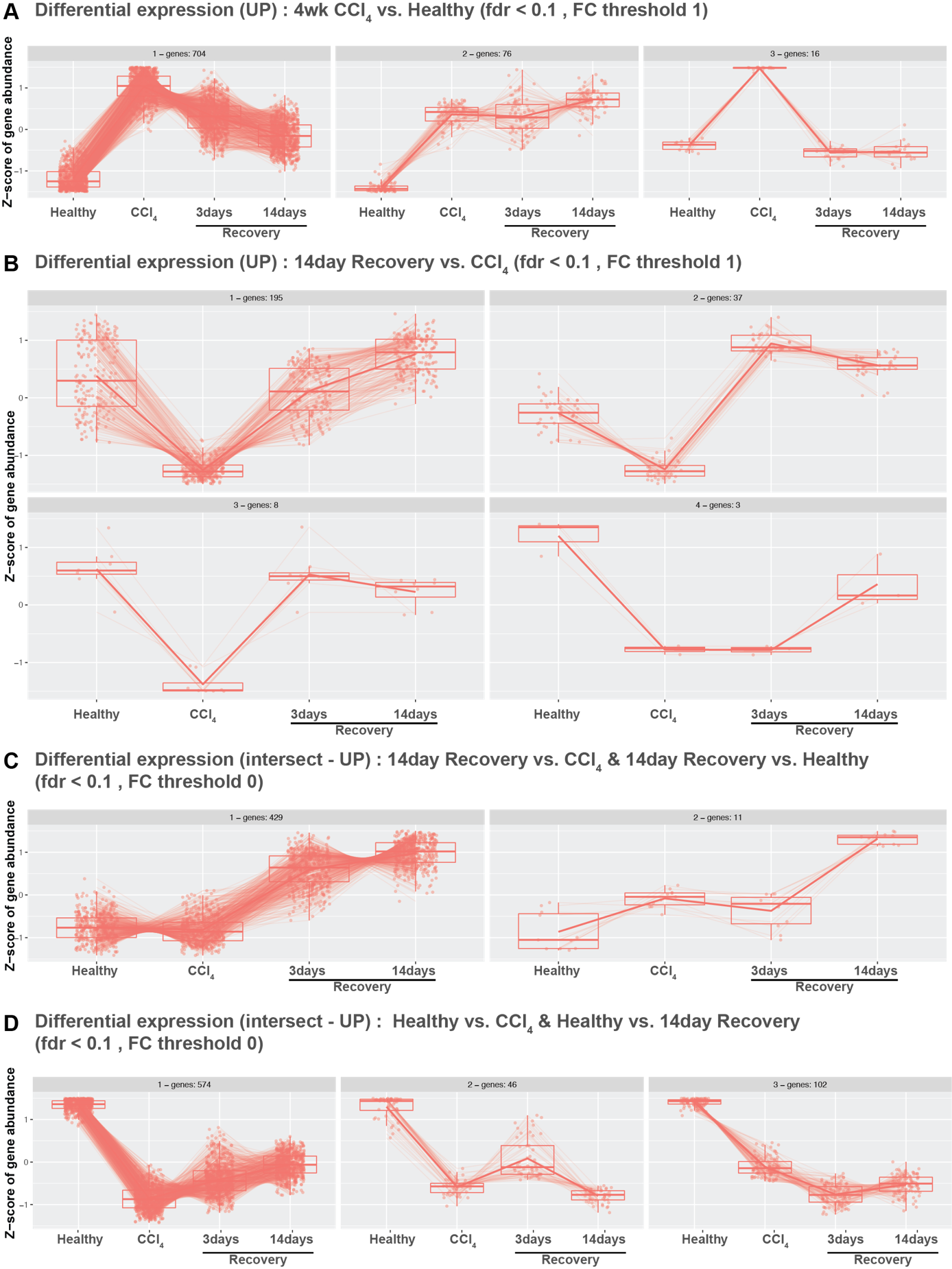


**Supplementary Fig. 1. Differentially expressed gene patterns.** (A) Patterns derived from genes upregulated in HSCs after 4weeks of CCl_4_ when compared to HSCs isolated from healthy mice. (B) Patterns derived from genes upregulated in HSCs after 2weeks of recovery from 4weeks of CCl_4_ when compared to HSCs isolated from mice subjected to 4weeks of CCl_4_. (C) HSC genes related to recovery of liver injury were determined by selecting common upregulated genes after 2weeks of recovery from 4weeks of CCl_4_ when compared to both HSCs isolated from healthy mice and mice subjected to 4weeks of CCl_4_. (D) HSC genes related to quiescence, were determined by selecting common upregulated genes in HSC isolated from a healthy liver when compared to both HSCs isolated from mice subjected to 4weeks of CCl_4_ and 2 week recovery from 4weeks of CCl_4_. All gene lists are available in Supplementary Table 1.

Supplementary Figure 2


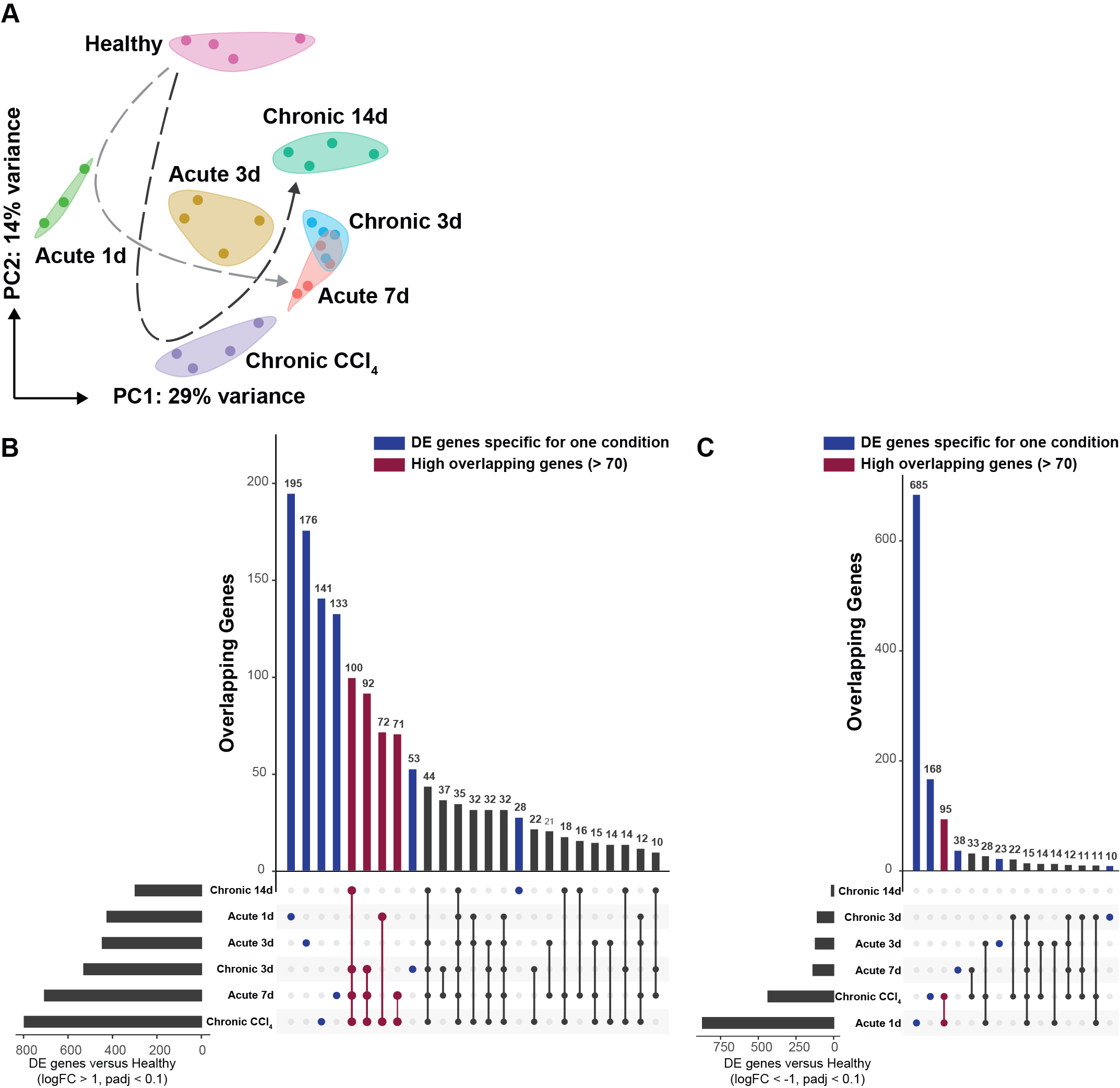


**Supplementary Fig.2. Global transcriptional alterations of HSCs following acute and chronic liver injury.** (A) Principal component analysis reveals distinct transcriptional dysregulation paths in HSCs following either acute (grey arrow) or chronic (black arrow) liver injury. (B-C) Upset plots of positive (B) or negative (C) differentially expressed genes (p < 0.1 and fold change > 2) between acute or chronic injured versus healthy livers. Genes that are enriched in one condition are represented in blue while overlapping genes (> 70 ) in red. Upset plots are created using R package UpSetR.

Supplementary Figure 3


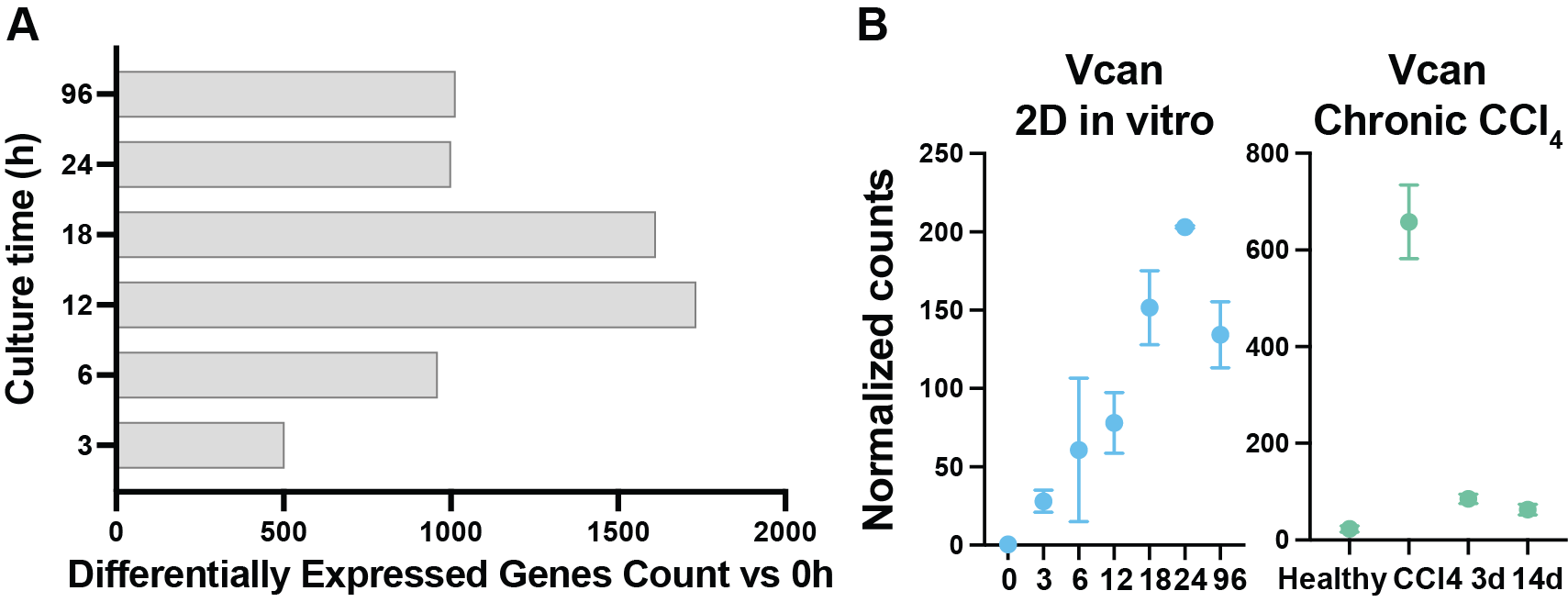


**Supplementary Fig.3. Transcriptional changes during in vitro HSC activation.** (A) Amount of differentially expressed genes in cultured HSCs when compared to freshly isolated HSCs. (B) Gene expression of Vcan as an example of similarities between HSC initiation and perpetuation.

Supplementary Figure 4


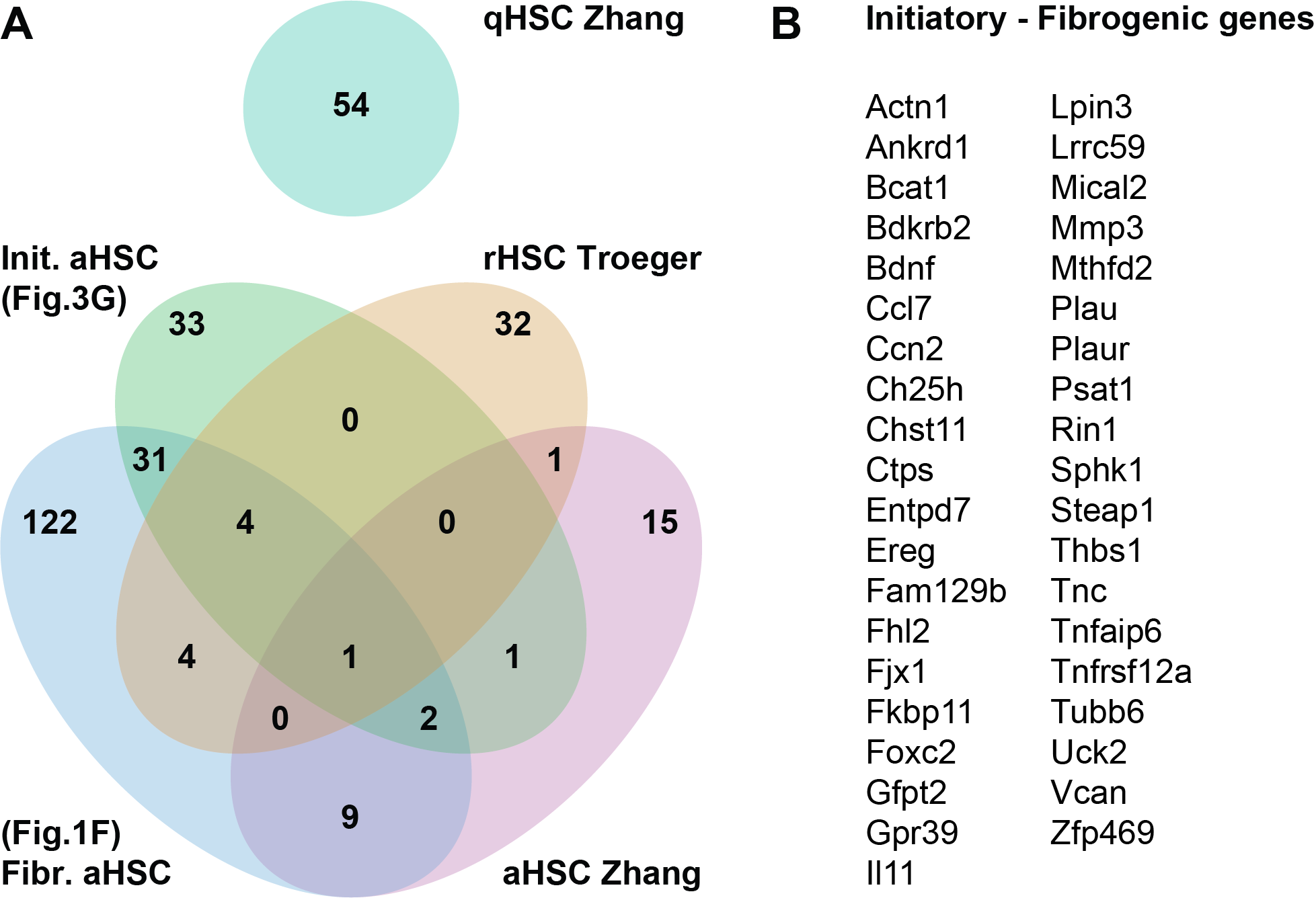


**Supplementary Fig. 4. Overview of selected HSC derived gene sets.** (A) Intersect of selected HSC derived gene sets as shown in Table 1. No genes in the qHSC signature overlap with activated or reverted gene sets. (B) Genes represented in both the initiatory aHSC and fibrogenic aHSC programs.

Supplementary Figure 5


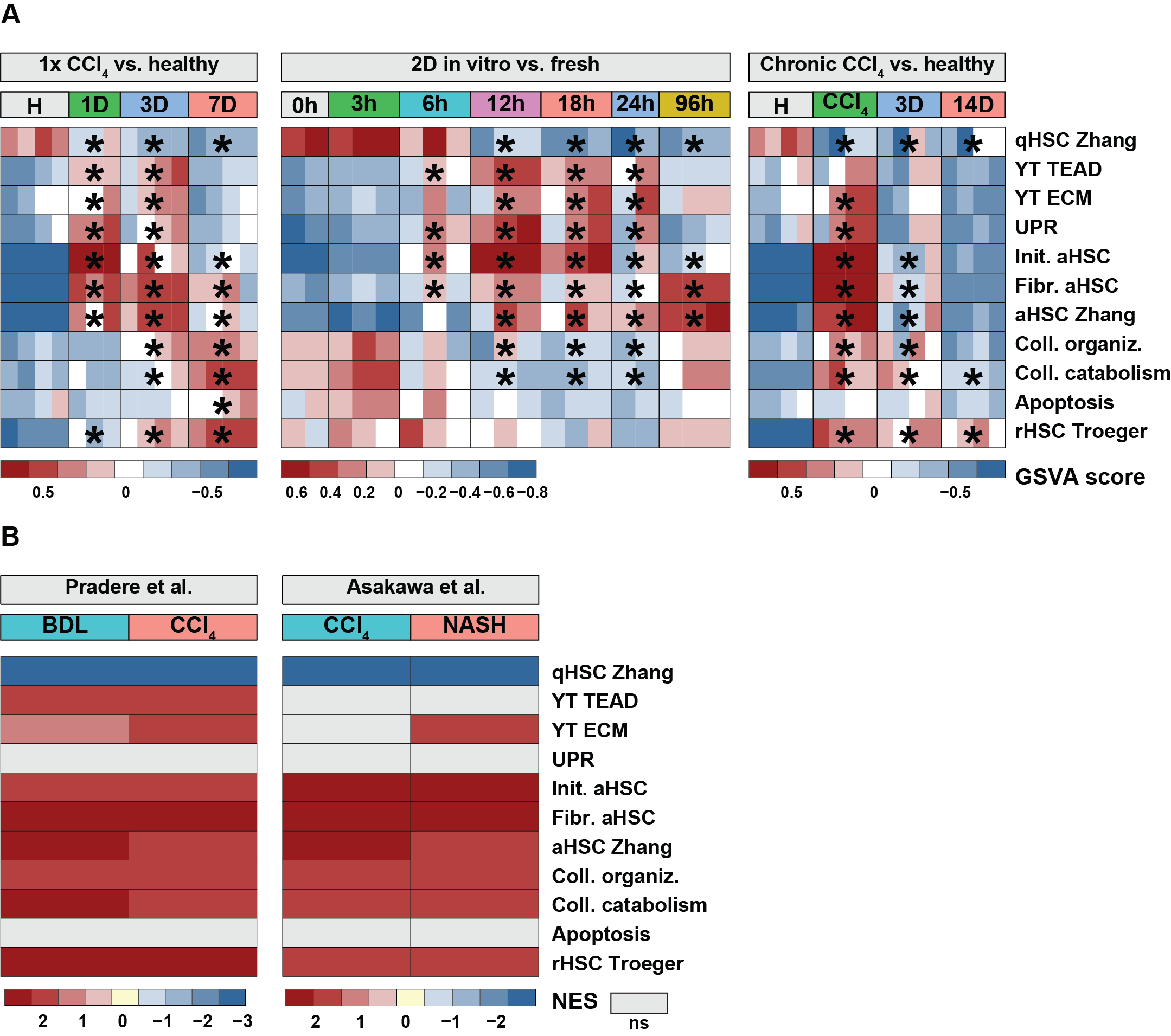


**Supplementary Fig. 5. Gene set variation and enrichment analysis of HSCs in independent models of HSC transcriptional dysregulation.** (A) GSVA on different models of HSC activation using gene sets defined in Table 1 and bulk RNAseq data from this study. Left panel: 1x CCl4. Middle panel: in vitro HSC activation. Right panel: 4 weeks CCl4 and recovery. * fdr < 0.05. (B) Validation of chronic liver injury results by performing GSEA on online available transcriptomic data of experimental liver fibrosis (BDL, NASH and CCl_4_)^1,2^.

**Supplementary Tables**

| **Study** | **Species** | **Transcriptome Platform** | **Data availability** | **Cell Type/Tissue** | **Synopsis** |
| --- | --- | --- | --- | --- | --- |
| Pradere et al,  2013 ^1^ | M | Affymetrix Mouse Genome 430 2.0 Array | GEO GSE34640 | HSC | ﻿C57BL/6 quiescent HSC and activated HSC following hepatotoxic (4 injections CCl_4_) and cholestatic (BDL – 15days) liver injury |
| Asakawa et al,  2019 ^2^ | M | Illumina Genome Analyzer IIx | GEO ﻿GSE134512 | HSC | Col1a2-GFP Tg quiescent HSC and activated HSC following hepatotoxic injury induced by either NASH (﻿MC4R-KO cross + 20wk WD) or CCl_4_ (semiweekly for 8 weeks) |
| Moylan et al,  2014 ^3^ | H | Affymetrix Human Genome U133 Plus 2.0 Array | GEO GSE49541 | Liver | Comparison of liver tissue from NAFLD patients with either F0-F1 (n = 40) or F3-F4 (n = 32) |
| Trepo et al,  2018 ^4^ | H | Affymetrix Human Genome U219 Array | GEO GSE103580 | Liver | Comparison of liver tissue from patients with various stages of ALD: simple steatosis (n = 6), mild acute alcoholic hepatitis (n = 13) and cirrhosis (n = 67) |
| Wang et al,  2018 ^5^ | H | Affymetrix Human Genome U133 Plus 2.0 Array | GEO GSE84044 | Liver | Comparison of liver tissue from patients with chronic HBV infection. For this manuscript, samples were divided into either ﻿Scheuer fibrosis score S0 or S1 (F0-F1 in this manuscript – n = 63) or Scheuer fibrosis score S2, S3 or S4 (F2-F4 in this manuscript – n = 61). |
| Wurmbach et al,  2007 ^6^ | H | Affymetrix Human Genome U133 Plus 2.0 Array | GEO GSE6764 | Liver | Comparison of liver tissue from patients with healthy livers (n = 10) or patients with chronic HCV infection induced cirrhosis: with concurrent HCC (n = 10) or without concurrent HCC (n = 3) |
| Krenkel et al,  2019 ^7^ | M | 10X Chromium - Illumina NextSeq 500 | GEO GSE132662 | HSC | Single cell RNA Sequencing of C57BL/6J quiescent HSC and activated HSC following hepatotoxic (9 injections CCl_4_ over 3 weeks) liver injury |
| Ramachandran et al,  2019 ^8^ | H | 10X Chromium - Illumina HiSeq 4000 | GEO GSE136103 | Epithelia, Immune, Endothelia and Mesenchyme | Single cell RNA Sequencing comparison of liver tissue from patients with healthy livers (n = 5) or patients with cirrhosis originating from NAFLD (n = 2), ALD (n = 2) or PBC (n = 1) |

**Supplementary Table 1. Transcriptome data sets analyzed in this manuscript.** ALD: alcoholic liver disease, BDL: bile duct ligation, CCl_4_: carbon tetrachloride, GEO: Gene Expression Omnibus (ncbi.nlm.nih.gov/geo), H: human, HBV: Hepatitis B virus, HCC: hepatocellular carcinoma, HCV: Hepatitis C virus, HSC: hepatic stellate cell, M: mouse, NAFLD: non-alcoholic fatty liver disease, NASH: non-alcoholic steatohepatitis, PBC: Primary biliary cholangitis, shRNA: short hairpin RNA, WD: western diet.

**Supplementary Table 2:** See file Supplementary Table 2­.xlsx

**Differentially expressed genes in experimental liver fibrosis and recovery**. (A) Genes upregulated in HSCs after 4weeks of CCl_4_ when compared to HSCs isolated from healthy mice. (B) Genes upregulated in HSCs after 2weeks of recovery from 4weeks of CCl_4_ when compared to HSCs isolated from mice subjected to 4weeks of CCl_4_. (C) HSC genes related to recovery of liver injury were determined by selecting common upregulated genes after 2weeks of recovery from 4weeks of CCl_4_ when compared to both HSCs isolated from healthy mice and mice subjected to 4weeks of CCl_4_. (D) HSC genes related to quiescence were determined by selecting common upregulated genes in HSC isolated from a healthy liver when compared to both HSCs isolated from mice subjected to 4weeks of CCl_4_ and 2 week recovery from 4weeks of CCl_4_.

**Supplementary Table 3:** See file Supplementary Table 3.xlsx

**A fibrogenic aHSC program based on the CCl_4_ model of liver fibrosis and reversal.** Differentially expressed genes in HSCs isolated from CCl_4_ treated mice compared to HSCs isolated from both healthy and recovered mice.

**Supplementary Table 4:** See file Supplementary Table 4.xlsx

**Global transcriptional alterations of HSCs following acute and chronic liver injury.** Genes that are enriched in one condition (represented in blue) and overlapping genes (> 70, represented in red) in the Upset plots of Suppl. Fig 2B and C. are listed in this xlsx.

**Supplementary Table 5:** See file Supplementary Table 5.xlsx

**Differentially expressed genes in experimental acute liver injury**. Genes upregulated in HSCs 24 hours after a single injection of CCl_4_ when compared to HSCs isolated from healthy mice.

**Supplementary Table 6:** See file Supplementary Table 6.xlsx

**An in vitro and in vivo conserved initiatory aHSC transcriptional program.** The initiatory aHSC program was generated by intersection “early in vivo” and “early in vitro” genes.

**References to Supplementary Data**

1. Pradere J-P, Kluwe J, De Minicis S, et al. Hepatic macrophages but not dendritic cells contribute to liver fibrosis by promoting the survival of activated hepatic stellate cells in mice. *Hepatology*. 2013;58(4):1461-1473. doi:10.1097/OPX.0b013e3182540562.The

2. Asakawa M, Itoh M, Suganami T, et al. Upregulation of cancer-associated gene expression in activated fibroblasts in a mouse model of non-alcoholic steatohepatitis. *Sci Rep*. 2019;9(1):1-14. doi:10.1038/s41598-019-56039-0

3. Moylan CA, Pang H, Dellinger A, et al. Hepatic Gene Expression Profiles Differentiate Presymptomatic Patients With Mild Versus Severe Nonalcoholic Fatty Liver Disease. *Hepatology*. 2014;59(2):471-482. doi:10.1002/hep.26661

4. Trépo E, Goossens N, Fujiwara N, et al. Combination of Gene Expression Signature and Model for End- stage Liver Disease Score Predicts Survival of Patients With Severe Alcoholic Hepatitis. *Gastroenterology*. 2018;154(4):965-975. doi:10.1053/j.gastro.2017.10.048.Combination

5. Wang M, Gong Q, Zhang J, et al. Characterization of gene expression profiles in HBV-related liver fibrosis patients and identification of ITGBL1 as a key regulator of fibrogenesis. *Sci Rep*. 2017;7(November 2016):1-13. doi:10.1038/srep43446

6. Wurmbach E, Chen YB, Khitrov G, et al. Genome-wide molecular profiles of HCV-induced dysplasia and hepatocellular carcinoma. *Hepatology*. 2007;45(4):938-947. doi:10.1002/hep.21622

7. Krenkel O, Hundertmark J, Ritz TP, Weiskirchen R, Tacke F. Single Cell RNA Sequencing Identifies Subsets of Hepatic Stellate Cells and Myofibroblasts in Liver Fibrosis. *Cells*. 2019;8(5):1-10.

8. Ramachandran P, Dobie R, Wilson-Kanamori JR, et al. Resolving the fibrotic niche of human liver cirrhosis at single-cell level. *Nature*. 2019;575(7783):512-518. doi:10.1038/s41586-019-1631-3
